# Hospital Meetings

**Published:** 1907-04-06

**Authors:** 


### HOSPITAL MEETINGS.

### MOUNT VERNON HOSPITAL FOR CONSUMPTION.

The annual meeting of the Court of Governors of the Mount Vernon Hospital was held on Wednesday afternoon at the offices of the hospital in Fitzroy Square. The chair was taken by Mr. J. S. Fletcher, M.P. for Hampstead, in the absence of the Marquis of Zetland, from whom a letter was read, regretting his inability to attend owing to an unexpected meeting in Yorkshire on the same date.

The report having been taken as read, Mr. Fletcher drew attention to the fact that the hospital could really congratulate itself this year. A special grant of £4,500 by the Council of King Edward's Hospital Fund, donations by Mr. Herbert Marnham, Mrs. Wharrie, and others, had released the committee from all their liabilities on the Building Fund account, and they were glad to be able to report that, with the exception of a mortgage of £2,500 upon their freehold property in Fitzroy Square, the hospital was free from encumbrances. The subscriptions, too, had increased from £2,909 in 1905 to £3,078 in 1906. On the other hand, there was accommodation for many more beds both at Hampstead and Northwood, but the committee were unable to open them owing to lack of funds. The demand on the part of patients was increasing, and the committee solicited further help to enable them to open the remaining beds. It might be pointed out that the additional expenses so incurred would only be for maintenance. In response to their appeal for £100,000, the Zuntz Estate Trustees had made a donation of £5,000, and promised an additional £15,000 on condition of the £100,000 being raised, and an anonymous donor promised a similar conditional donation of £20,000, so that they had, with investments from legacies, not far short of half the amount required for opening the additional beds available. The committee much regretted to record the deaths of Mr. W. S. Gard, Mr. Frederick Claudet, Mrs. Cash, Miss Cloves Carter, and Mr. Alfred Beit, all of whom generously assisted the hospital.

Mr. Henry Stedall, the Chairman of the Committee of Management, in answer to a question of one of the governors with reference to the large increase in the number of out-patients, pointed out that the majority of them were sent by patients' medical attendants who knew that the hospital could provide special treatment. He would also like to say that every effort was made to prevent any but indigent persons from attending as out-patients. Dr. O'Connor was elected a Vice-President of the hospital, and Colonel Arthur, Mr. S. Baer, Mr. J. S. Fletcher, M.P., Dr. Pidcock, and Dr. Weaver were re-elected to the Committee of Management.

